# Personal

**Published:** 1892-09

**Authors:** 


### Personal.

DR. GEORGE L. BROWN announces his removal from No. 173 Niagara street to 121 Franklin street, Buffalo. His office hours are 9 to 11 A. M., 1 to 4 P. M. Sundays, 10 A. M. and 2 P. M. Residence, 204 Highland avenue.

---

DR. JOHN O. ROE, the well-known laryngologist, of Rochester, N. Y., has been invited by the faculty of the Tennessee Medical College, at Knoxville, to deliver a special course of four lectures in the department of laryngology and rhinology. Several distinguished specialists in other branches have also accepted similar invitations, which will add to the interest of the Winter term in that institution. It is expected that Dr. Roe's lectures will be delivered during February, 1893, and they will be of a practical nature.

Dr. Bransford Lewis, editor of the *Medical Fortnightly*, has recently been appointed Consultant in Genito-Urinary Surgery to the City Hospital, the Female Hospital, the Missouri Pacific Hospital, and St. Mary's Infirmary, in St. Louis. Dr. Lewis is well fitted for these honorable stations.

---

Dr. W. E. B. Davis, whose removal to Rome, Ga., we chronicled in the February number of the Journal, has returned to Birmingham, Ala., and again taken up his practice there. The Davis brothers are renowned for their excellent surgical and gynecological work, and in keeping their interests united are acting for the best interests of a large region of country, of which Birmingham is the natural center.

